# RFX1 Regulates Immune Microenvironment and Predicts Immunotherapy Response in Colon Cancer: A Multi-Omics and Clinical Analysis

**DOI:** 10.32604/or.2025.068473

**Published:** 2025-11-27

**Authors:** Zhujiang Dai, Xiaoyong Ge, Wenbo Tang, Chen-Ying Liu, Yun Liu, Zhongchuan Wang

**Affiliations:** 1 Department of Colorectal Surgery, Xinhua Hospital, Shanghai Jiaotong University School of Medicine, Shanghai, 200092, China; 2 Shanghai Colorectal Cancer Research Center, Shanghai, 200092, China

**Keywords:** Colon cancer, regulatory factory X1, tumor microenvironment, persistent tumor mutational burden, pan-cancer

## Abstract

**Objective:**

The plastic role of regulatory factor X1 (RFX1) in colon cancer progression and its impact on the tumor microenvironment remain poorly understood. The study aimed to clarify the molecular and clinical role of RFX1 in colon cancer.

**Methods:**

We classified colon cancers into subgroups with high and low RFX1 expression and characterized their immune profiles, mutational profiles, cancer immunotherapy and drug sensitivity. By combining RFX1 expression with persistent tumor mutational burden, we proposed a novel nomogram clinical prediction model and validated its predictive performance, and the correlation between high expression and poor prognosis.

**Results:**

Compared to tumor mutational burden (TMB), persistent tumor mutational burden (pTMB) is an independent predictor of prognosis in patients with colon cancer. The predictive efficacy of the combination of RFX1 expression and pTMB was superior to and sensitive than the combination of RFX1 expression with TMB. Among them, patients in the RFX1^high^/pTMB^high^ subgroup had the worst quality of survival and prognosis, whereas those in the RFX1^low^/pTMB^low^ subgroup had a relatively better prognosis (*p* < 0.0001). Univariate Cox regression revealed a significant association between high RFX1 expression and increased risk in colon cancer patients (Hazard Ratio [HR] = 1.58, 95% Confidence Interval [CI]: 1.10–2.25, *p* = 0.012), which remained independently predictive in multivariate analysis after covariate adjustment (HR = 1.52, 95% CI: 1.04–2.22, *p* = 0.031).

**Conclusion:**

A nomogram model based on RFX1 combined with pTMB provides an alternative approach for the diagnosis and treatment of colon cancer.

## 1 Introduction

Colon cancer is gradually becoming a major burden on the global public health system with its high incidence and diagnosis rates [1]. The clinical symptoms of colon cancer are often overlooked, resulting in a poor prognosis for patients in advanced stages. In recent years, the malignant lesions of the colon have gradually become younger, which may be attributed to the irregular diet and work habits of young people [2]. In addition, persistent accumulation of genetic and epigenetic inheritance forces heterogeneous proliferation of the intestinal epithelium, which progresses to a malignant phenotype [3]. Advances in genomics, proteomics, and molecular pathology have led to several candidate biomarkers with potential clinical utility for cancer staging, genomic, epigenomic, and/or immunological analyses for selective therapies, and for monitoring patient prognosis [4]. Although biomarkers for colon cancer have been widely used for tumor screening and diagnosis, most detection techniques often struggle to balance sensitivity and specificity. For example, specific gene mutations (KRAS, APC, BRAF) lack sufficient sensitivity and make it difficult to confirm the presence of tumors [5]. Although circulating tumor DNA is novel for identifying tumor heterogeneity, its hypomethylation makes it difficult to accurately predict tumor metastasis accurately [6,7]. Therefore, the search for robust and broad-spectrum tumor markers remains a challenge.

Regulatory factor X1 (RFX1), a conserved transcription factor, assumes a paramount role in orchestrating the intricate choreography of cellular growth and development [8]. This includes precise governance of fundamental cellular activities, such as division, proliferation, differentiation, and the finely orchestrated mechanism of apoptosis. RFX1 belongs to the RFX gene family, and has an evolutionarily conserved wing-helical DNA-binding domain (DBD) that binds to X-box motifs in target DNA [9,10]. In addition, RFX1 has a unique C-terminal repressor domain and an N-terminal glutamine-rich activation domain (AD), which together regulate its pleiotropic behavior [11]. The nuclear localization of RFX1 is dependent on strong nuclear localization signals in the C-terminal repressor domain and weak nuclear localization signals within the DBD, and is negatively regulated by the C-terminal acidic stretch [9].

Evidence indicates that RFX1 is differentially expressed in most tumors [10]. In an array of diverse cancer tissues, including those of the breast, bone, lung, esophagus, prostate, and an additional 11 tissues, a notable and statistically significant reduction in RFX1 expression was observed [10,12]. Conversely, heightened expression levels of RFX1 were discerned in ovarian, pancreatic, hepatic, and an additional ten tissue types [10]. In contrast, the expression levels of RFX1 exhibited no statistically significant alterations when scrutinized in the context of brain, kidney, and vulvar cancer tissues, as compared to their normal tissue counterparts [10]. Furthermore, RFX1 functions as a transcriptional repressor, exerting regulatory influence on several oncogenes, including c-Myc, FGF1, TGF-β2, COL1A1, COL1A2, CDX2, and TLR4 [13,14]. Its anti-tumor efficacy is evident by a conspicuous reduction in RFX1 expression levels observed during the transition from normal epithelial cells to adenocarcinomas, as demonstrated by tissue sample analyses [15]. RFX1-mediated tumor suppression can also be mediated by inducing cancer stem cell differentiation, thereby rendering cancer cells vulnerable to chemotherapy [16].

In the realm of immune system modulation, RFX1 assumes a dual role: an activator of MHC class II genes and a suppressor of MCP1, CD11a, CD70, IL17A, TLR4, and TGFβ2 expressions [17–19]. This multifaceted involvement not only enhances our comprehension of cancer etiology and immune-related disorders but also paves the way for the conceptualization of innovative therapeutic strategies. A Recent work by Yang et al. further revealed a critical role for RFX1 in inflammatory bowel disease: RFX1 promotes M1 macrophage polarization by transcriptionally activating APOBEC3A, which induces DNA demethylation of key inflammatory cytokines such as IL6 and TNF [19]. In mouse models, RFX1 deficiency or pharmacological inhibition significantly reduced tissue inflammation and damage, highlighting its immunopathogenic potential and therapeutic relevance. So far, few have systematically analyzed its association with the tumor immune microenvironment, mutational burden dynamics, and immunotherapy response, especially in the context of colon cancer. We have gradually focused our attention from pan-cancer to the molecular characterization of RFX1 in colon cancer. We classified colon cancers into subgroups with high and low RFX1 expression and characterized their immune profiles, mutational profiles, tumor immunotherapy and drug sensitivity.

In addition, we not only elucidated the expression and prognostic value of RFX1 in colon cancer using multi-omics analysis, but also innovatively introduced persistent tumor mutational burden (pTMB) as a co-stratification tool. Furthermore, we constructed a novel RFX1-pTMB-based nomogram model for clinical prediction and validated its performance through external clinical samples and drug sensitivity correlation analysis. These findings provide insights into the immunogenomic and therapeutic relevance of RFX1 in colon cancer.

Collectively, these findings raise the hypothesis that RFX1 acts as an oncogenic transcription factor in colon cancer by shaping an immunosuppressive microenvironment, modulating mutational dynamics, and conferring resistance to immunotherapy. To test this hypothesis, we integrated transcriptomic, mutational, and pharmacogenomic data to delineate the multifaceted role of RFX1 in tumor progression and therapy response. Our aim is to determine whether RFX1 can serve as a predictive biomarker and potential target in the immunogenomic stratification of colon cancer.

## 2 Materials and Methods

### 2.1 Data Initial Resources

Expression profiles and patient follow-up information for 33 cancer types were obtained from The Cancer Genome Atlas (TCGA) database (https://portal.gdc.cancer.gov/) (accessed on 17 August 2025). For the mutation data, we performed the visualisation of the Maftools [20] tool (v2.14.0) based on the preprocessing results of mutect [21]. The raw data from the three datasets GSE17538, GSE33113 and GSE38832 were standardised and de-batched to remove the batch effect, and then we combined them into one dataset GSE [22]. Batch effects among the three datasets were corrected using the ComBat function from the “sva” package (v3.44.0). Afterwards, the data were merged into a single integrated dataset for subsequent analysis. Probe annotations were mapped to gene symbols based on the GPL570 platform: for multiple probes corresponding to the same gene, the median expression was used; probes mapping to multiple genes were excluded. Only colorectal cancer tumor samples with available survival time and status were retained for downstream analysis. The cleaning and merging of the three Gene Expression Omnibus (GEO; https://www.ncbi.nlm.nih.gov/geo/) (accessed on 17 August 2025) datasets were elaborated step by step in the previous work [23].

### 2.2 Principal Component Analysis (PCA)

Principal Component Analysis (PCA) was performed to reduce data dimensionality while preserving the majority of variance. PCA transforms original correlated variables into a set of uncorrelated principal components. The analysis was conducted using R software (v 4.3.0) with the “FactoMineR” package (v 2.8) and visualized using the “factoextra” package (v 1.0.7).

### 2.3 Kaplan-Meier (KM) Survival Analysis and Visualization

KM analysis is a widely used non-parametric method for estimating the survival function based on time-to-event data, effectively accounting for censored observations such as loss to follow-up or study termination. In this study, KM curves were employed to compare survival differences between patient subgroups stratified by RFX1 expression levels. Survival curves were generated and visualized using the R package “survminer” (v 0.4.9). The log-rank test was used to assess statistical significance between groups, allowing for clear interpretation of survival trends.

### 2.4 Single-Sample Gene Set Enrichment Analysis (ssGSEA)

ssGSEA calculates enrichment scores for each sample individually, quantifying the activity of predefined gene sets. GSEA evaluates whether predefined gene sets show statistically significant differences between two groups, based on ranked gene expression data. The genetic signature of the pathway was characterised by us, supported by the R (v4.4.3) package “GSVA” (v1.50.1) and “GSEABase” (v1.50.1) [24], with statistical significance defined as *p* < 0.05 and false discovery rate (FDR) < 0.25 for enrichment analyses.

### 2.5 Correlation Analysis of RFX1 with Signature Genes in Pan-Cancer

We obtained gene expression profiles of chemokines, immunoinhibitors, MHC, receptors, and immune checkpoints, and then analysed the correlation between these signature genes and RFX1 in pan-cancer [25,26]. We calculated Pearson correlation coefficients between RFX1 expression and the expression levels of these immune-related signature genes to assess their linear relationships. To ensure robustness, *p*-values were adjusted using the Benjamini-Hochberg method to control the false discovery rate (FDR) due to multiple comparisons. Genes with adjusted *p*-values below 0.05 were considered significantly correlated with RFX1. We further analysed the correlation between immune cell scores and gene RFX1 in pan-cancer based on the signature gene scores of immune cells [27].

### 2.6 pTMB

pTMB was calculated following the approach described by Niknafs et al. in Nature Medicine [28]. Briefly, pTMB quantifies the number of somatic mutations that are likely to be retained across tumor evolution, specifically those located in single-copy (haploid) or multi-copy genomic regions that are less likely to be lost. These “persistent mutations” provide a distinct measure from total TMB, as they remain through selective pressure [29]. We intersected nonsynonymous somatic mutations with copy number profiles using the “maftools” R package (v2.14.0), and defined pTMB as the count of mutations in haploid or amplified regions per sample. This approach ensures pTMB reflects stable mutational signals rather than transient mutational events. The waterfall plot is used here to visually display the distribution and frequency of gene mutations across different subgroups. It allows clear identification of differential genes with high mutation burdens, helping to highlight key mutational drivers and potential associations between gene mutations and RFX1 expression levels.

### 2.7 Predicting the Benefits of Immunotherapy and Targeted Therapy in the RFX1^***high***^ and RFX1^***low***^ Subgroups

The technical concepts and steps related to the SubMap analysis have been explained in detail in our previous articles [30]. The method indirectly predicts subgroup response to immunotherapy by fitting similarities in gene expression profiles between subgroups and immunotherapy groups [31,32]. We harnessed the predictive power of the R package “pRRophetic” (v0.5) to anticipate drug sensitivity in relation to IC_50_ values and to ascertain drugs that exhibit significant associations with RFX1 [33].

### 2.8 Tumor Immune Dysfunction and Exclusion (TIDE) Predicts Response

The technical pathways associated with TIDE have been thoroughly elucidated and substantiated in our prior research endeavors [23,34,35]. The TIDE database (http://tide.dfci.harvard.edu/) (accessed on 17 August 2025) is a computational platform developed to model tumor immune evasion mechanisms based on gene expression data. It integrates multiple public immunotherapy cohorts with clinical annotation and provides predictive scores, including TIDE score, microsatellite instability (MSI) status, dysfunction score, and exclusion score. In our study, we utilized TIDE to evaluate the association between RFX1 expression and predicted response to immune checkpoint inhibitors (ICIs) across a large pan-cancer dataset. This approach allows for broad validation of immunotherapy-related biomarkers without being limited to a single tumor type. The TIDE framework (http://tide.dfci.harvard.edu/), which stands for tumor immune dysfunction and exclusion, represents a computational methodology employed for evaluating the probability of immune escape within gene expression profiles of tumor samples, as expounded upon in our earlier studies.

### 2.9 Presentation of the Nomogram Model

The nomogram model was comprehensively utilized to integrate tumor clinical stage, RFX1 expression, and pTMB, enabling the evaluation of the overall survival of patients at one, three, and five-year intervals.

### 2.10 Cell Culture and Transfection

Human CRC cell lines HCT116, SW-480 and LoVo were all purchased from the American Type Culture Collection (ATCC; Manassas, VA, USA). LoVo was cultured in Kaighn’s Modification of Ham’s F-12 Medium (F12-K) (Gibco, C11765500BT, Grand Island, NY, USA), and the other cell lines were cultured in Dulbecco’s Modified Eagle’s Medium (DMEM) (Hyclone, SH30022, Logan, UT, USA) all supplemented with 10% fetal bovine serum (Gibco, Fetal Bovine Serum, Premium Plus A5669701) and 1% penicillin/streptomycin at 37°C with 5% CO_2_. All cell lines were authenticated by short tandem repeat profiling using GenePrint^®^ 10 System (Promega, Madison, WI, USA). Mycoplasma testing is routinely performed monthly in our laboratory to avoid mycoplasma contamination. Stable RFX1 knockdown cell lines, shRFX1-1 and shRFX1-2, were engineered through the deployment of the pLKO.1 vector system. To construct stable knockdown cell lines, oligonucleotides encoding shRFX1-1 and shRFX1-2 sequences (Biosharp, Shanghai, China) were synthesized, annealed, and subsequently ligated into the pLKO.1 vector (Novopro, V015125, Shanghai, China) digested with AgeI and EcoRI restriction enzymes. The ligation products were transformed into competent *E. coli* DH5α cells, and positive clones were selected and confirmed by Sanger sequencing. Verified plasmids were co-transfected with packaging plasmids psPAX2 (Addgene, 12260, Beijing, China) and pMD2.G (Addgene, 12259) into HEK293T cells using Lipofectamine 2000 (Invitrogen, 11668019, Carlsbad, CA, USA) to produce lentiviral particles.

### 2.11 Western Blot

The human tumor samples were collected between June 2008 and December 2018 at Xinhua Hospital, which is affiliated with Shanghai Jiaotong University School of Medicine. The ten tumor samples, which also included paracancerous tissues, were sourced from the primary cohort featured in this paper and received informed consent from all participants or their legal guardians and ethical approval from the Ethics Committee of Shanghai Xinhua Hospital (XHEC-C-2024-206-1). Fresh patient tissue samples were first flash-frozen in liquid nitrogen and stored at −80°C until use. For protein extraction, approximately 50 mg of tissue was placed into a 2 mL microcentrifuge tube containing stainless steel beads and 500 µL of ice-cold RIPA lysis buffer (Beyotime, P0013B, Shanghai, China) supplemented with protease and phosphatase inhibitors. The tissue was homogenized using a bead mill homogenizer (TissueLyser II, Qiagen) at 30 Hz for 3 cycles of 1 min each, with 1-min intervals on ice between cycles to prevent protein degradation. The homogenate was then centrifuged at 12,000 rpm for 15 min at 4°C, and the supernatant containing total protein was collected. Protein concentration was determined using a BCA protein assay kit (Beyotime, P0010). Equal amounts of protein (20 µg per lane) were loaded onto 10% SDS-PAGE gels for electrophoresis. After separation, proteins were transferred onto NC membranes (Cytiva, 10600002, Marlborough, MA, USA) using a wet transfer system at 300 mA for 60 min at 4°C. Membranes were blocked with 5% non-fat dry milk dissolved in Tris-buffered saline with 0.1% Tween-20 (TBST) for 1 h at room temperature to prevent non-specific binding. Subsequently, membranes were incubated overnight at 4°C with primary antibodies diluted in blocking buffer. After washing three times with TBST, membranes were incubated with HRP-conjugated secondary antibodies for 1 h at room temperature. Detection was performed using an enhanced chemiluminescence (ECL) kit (Beyotime, P0018S). The protein extraction procedures for HCT116, SW-480, and LoVo cell lines were identical to those described above. All experiments were performed in at least three independent biological replicates to ensure reproducibility. Each Western blot assay included technical duplicates. The protein extraction procedures for HCT116, SW-480, and LoVo cell lines were identical to those described above. For this study, we employed the following antibodies: RFX1 (26859, Proteintech, diluted at 1:1000 for Western Blot and 1:100 for Immunohistochemistry, Wuhan, China) and β-actin (A2228, Sigma-Aldrich, diluted at 1:10,000 for Western Blot, St. Louis, MO, USA).

### 2.12 Reverse Transcription Quantitative Polymerase Chain Reaction

The ten tumor samples, which included paracancerous tissues, were sourced from the initial unit of this study and received official authorization and ethical approval from the Ethics Committee of Shanghai Xinhua Hospital. All patients received comprehensive instructions and granted informed consent as part of the study process. Total RNA was extracted from freshly frozen human tumor tissues using a bead homogenization method. Approximately 30–50 mg of tissue was placed into a 2 mL RNase-free microcentrifuge tube containing stainless steel beads and 1 mL of TRIzol reagent (Invitrogen, 15596026CN, Carlsbad, CA, USA). The tissue was homogenized using a bead mill homogenizer (TissueLyser II, Qiagen) at 30 Hz for 3 × 1-min cycles, with cooling on ice between cycles to prevent RNA degradation. The homogenates were incubated at room temperature for 5 min, followed by the addition of 200 μL chloroform. After vigorous shaking and centrifugation at 12,000 rpm for 15 min at 4°C, the aqueous phase was transferred to a new RNase-free tube, and RNA was precipitated with isopropanol. The RNA pellet was washed with 75% ethanol, air-dried, and resuspended in RNase-free water. RNA concentration and purity were determined using a NanoDrop spectrophotometer (Thermo Fisher Scientific, Waltham, MA, USA). 500 ng of total RNA was reverse-transcribed into complementary DNA (cDNA) using the HiScript III 1st Strand cDNA Synthesis Kit (Vazyme, R323-01, Nanjing, China). qPCR was performed using SYBR Green Master Mix (Vazyme, Q711) on Applied Biosystems 7500 instrument (Thermo Fisher Scientific Inc, Waltham, MA, USA). Each reaction was carried out in a final volume of 10 μL, containing 5 μL of SYBR Green mix, 0.4 μL of forward and reverse primers, 4.6 μL of diluted cDNA. The thermal cycling conditions were as follows: initial denaturation at 95°C for 30 s, followed by 40 cycles of 95°C for 5 s and 60°C for 30 s. Relative gene expression levels were calculated using the 2^–ΔΔCt^ method, with GAPDH as the internal control. All experiments were conducted in triplicate with at least three independent biological replicates. The RNA extraction procedures and RT-qPCR experiments for the three cell lines HCT116, SW-480, and LoVo were conducted as described above. The PCR primer sequences utilized in this investigation are as follows, including RFX1 (forward: 5^′^-TTATTCAGCTCGCTGGCTTTG-3^′^, reverse: 5^′^-CAGCTTGGAGAAGTTCACGACG-3^′^) and GAPDH (forward: 5^′^-GTCTCCTCTGACTTCAACAGCG-3^′^, reverse: 5^′^-ACCACCCTGTTGTTGCTGTAGCCAA-3^′^).

### 2.13 Human Tissue Samples and Immunohistochemistry (IHC)

Human colorectal cancer specimens were collected within the timeframe spanning from August 2008 to November 2018, and subsequent postoperative follow-up data were meticulously documented until August 2020. Tumor tissues fixed in 4% paraformaldehyde were processed for paraffin embedding and sectioning (4 μm thickness) by Ruiyu Biotechnology Co., Ltd. (Shanghai, China). For immunohistochemical analysis, tissue sections were first baked at 65°C for 2 h to melt residual paraffin. Slides were then deparaffinized in xylene and rehydrated through a graded ethanol series (100%, 95%, 75%) to water. After washing in running tap water for 10 min, the slides were rinsed in phosphate-buffered saline (PBS) three times on a shaker (5 min each). Antigen retrieval was performed using 1× sodium citrate buffer (pH 6.0) (Sangon Biotech, E673001, Shanghai, China) in a microwave oven, with two 10-min heating cycles separated by a 5-min cooling period. After cooling to room temperature, slides were washed three times in PBS. Endogenous peroxidase activity was blocked by incubation in 3% hydrogen peroxide (prepared from 30% H_2_O_2_ diluted in methanol) for 15 min in the dark. Following further PBS washes, non-specific binding was blocked by incubating tissue sections with 5% normal goat serum (Beyotime, C0265) at room temperature for 1 h. Primary antibodies were diluted 1:100 in 5% goat serum and applied to the tissue area outlined with a hydrophobic barrier pen. Slides were incubated in a humidified chamber at 4°C overnight. The next day, after equilibration to room temperature, slides were washed in PBS and incubated with a universal HRP-conjugated secondary antibody (GeneTech, Shanghai, China) for 1 h at room temperature. DAB substrate (A:B = 1:50) (GeneTech, GK600705) was applied to develop color, and the reaction was monitored under a microscope to control staining time. The slides were then rinsed in running water for 5 min. Hematoxylin was used to counterstain nuclei for 60 s, followed by thorough washing in water and PBS. Dehydration was carried out through graded ethanol (75%, 95%, 100%) and xylene. Finally, slides were mounted with neutral resin and sealed with coverslips. Immunohistochemical staining results were evaluated independently by two pathologists, and RFX1 expression was categorized into “low” and “high” based on the Immunoreactivity Score (IRS) criteria, where IRS < 6 denoted low expression and IRS ≥ 6 indicated high expression. Rigorous measures were taken to secure ethics committee approval and obtain informed consent from all relevant parties.

### 2.14 Small Interfering RNA (siRNA) Transfection

The siRNA was purchased from Shanghai GenePharma Pharmaceutical Technology Company (Shanghai, China). The cell lines involved in this part of the experiment include HCT116, SW-480, and LoVo. Cells were seeded in 6-well plates at a density of 2 × 10^5^ cells per well and cultured overnight to reach approximately 60%–70% confluence at the time of transfection. For each well, 10 μL of siRNA and 10 μL of Rfect reagent (BAIDAI, 11012, Changzhou, China) were separately diluted in 100 μL of Opti-MEM™ Reduced Serum Medium (Gibco, 31985-070), incubated at room temperature for 5 min, then mixed and further incubated for 15–20 min to allow complex formation. The complexes were then added dropwise to the cells. Cells were transfected with RFX1 siRNA for 72 h and then collected for subsequent functional experiments. All experiments were performed in biological triplicates with appropriate controls.

### 2.15 Cell Function Experiments

The cell function experiments covered in this study mainly include Cell Counting Kit-8 (CCK8, Vazyme A311-01, Nanjing, China), transwell, clone formation, and scratch assays. The four functional experiments in this section involved three cell lines: HCT116, SW-480, and LoVo. For the transwell invasion assay, 1.5 × 10^5^ cells suspended in 250 μL of serum-free medium were seeded into the upper chambers of 8.0 μm pore size transwell inserts (Biofil, TCS020024, Guangzhou, China). The lower chambers were filled with 750 μL of complete medium containing 10% FBS. After 48 h of incubation at 37°C with 5% CO_2_, the non-migrated cells on the upper surface of the membrane were gently removed using a cotton swab. The migrated cells on the lower surface were fixed with 4% paraformaldehyde (Biosharp, BL539A, Shanghai, China) for 30 min and stained with 0.5% crystal violet solution (Solarbio, G1062, Beijing, China) for 15 min. After thorough washing with PBS, stained cells were imaged and counted under a microscope. The PBS used is a 1× solution with a pH of 7.3–7.5. It contains sodium chloride, potassium dihydrogen phosphate, and disodium hydrogen phosphate, providing a physiologically isotonic buffer environment. For the cell proliferation assay, 1000 cells per well were plated in 96-well plates (Corning, 3599, Corning, NY, USA) in sextuplicate. At the same time each day for five consecutive days, 10 μL of CCK-8 reagent was added to each well, followed by 2 h of incubation. Absorbance at 450 nm was measured using a microplate reader (BioTek, ELx800, Winooski, VT, USA) to assess cell viability. For the colony formation assay, 5000 cells per well were seeded into 6-well plates (Corning, 3516) and cultured under standard conditions for 10 to 14 days until visible colonies formed. Cells were then fixed in 4% paraformaldehyde for 30 min and stained with 0.5% crystal violet. After washing with water and air-drying, colonies were photographed and manually counted. For the wound healing assay, cells were seeded into 6-well plates and cultured to nearly 90–100% confluence. A sterile 200 μL pipette tip was used to create a straight scratch in the cell monolayer. Detached cells were removed by washing with PBS, and fresh serum-free medium was added. The plates were then incubated at 37°C in a 5% CO_2_ atmosphere. Wound closure was imaged at 0 and 24 h using an inverted microscope. The detailed steps for each part of the functional experiments have been described in our previous work. Finally, we applied Image J (v1.53t, National Institutes of Health, Bethesda, MD, USA) for imaging and counting.

### 2.16 Mouse Xenograft Tumor Model

All animal experiments in this study were approved by the Animal Care and Welfare Committee of Xinhua Hospital (XHEC-NSFC-2020-115). A total of 18 male BALB/c nude mice (aged 4–6 weeks) were purchased from Shanghai Jihui Laboratory Animal Company. The mice were housed in a specific pathogen-free (SPF) facility under controlled environmental conditions (temperature: 22 ± 2°C, humidity: 55 ± 10%, light/dark cycle: 12/12 h), with free access to standard chow and water. The mice were randomly divided into 3 groups (n = 6 per group) using a random number table. All procedures, tumor measurements, and endpoint analyses were conducted in a blinded fashion. The mice were randomly injected subcutaneously with 1,000,000 HCT-116 cells. Tumor size was measured every three days using a digital caliper, and tumor volume was calculated as (length × width^2^)/2. Mice were monitored daily for signs of distress, weight loss, or skin ulceration. At 14 days post-injection, mice were euthanized by CO_2_ inhalation followed by cervical dislocation in accordance with institutional ethical guidelines. Tumors were harvested, photographed, weighed, and processed for histological analysis.

### 2.17 Statistical Analysis

Data analysis in this study was performed using GraphPad Prism (v9, GraphPad Software, San Diego, CA, USA). All quantitative data are expressed as mean ± standard deviation (SD). The Wilcoxon signed-rank test was utilized for the IHC analysis of the Tissue Microarray (TMA), while Pearson correlation analysis assessed the statistical significance of mRNA levels from the TCGA dataset, and Spearman correlation analysis was applied to the IHC analysis of the TMA. The chi-square test is a non-parametric statistical method commonly used to assess whether there is a significant association between categorical variables. In this study, it was applied to analyze the relationship between RFX1 expression levels and various clinicopathological features. *p* < 0.05 was considered statistically significant.

## 3 Results

### 3.1 Data Preprocessing

**Figure A1:**
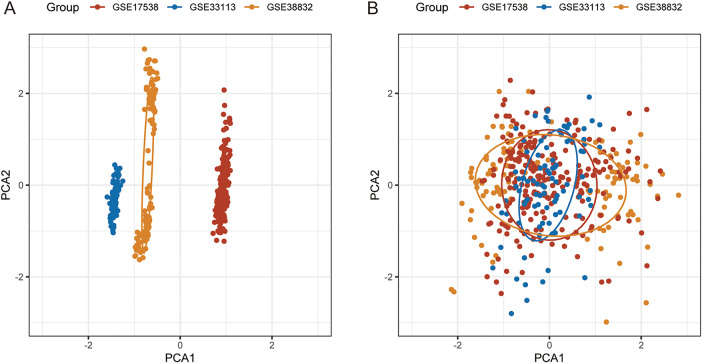
PCA analysis before and after batch effect correction. (**A**): PCA analysis prior to elimination of batch effects. (**B**): PCA analysis after elimination of batch effects

Based on the Combat function of the “sva” package, we eliminated the batch effect between GSE17538, GSE33113, and GSE38832 and merged them into the GSE dataset [36]. Before and after removing the batch effect, we performed PCA analyses on each dataset and found that the datasets were no longer significantly different. (Fig. A1A,B).

### 3.2 Immunological Landscape of RFX1 in Pan-Cancer and Clinical Prognosis in Colon Cancer

**Figure 1:**
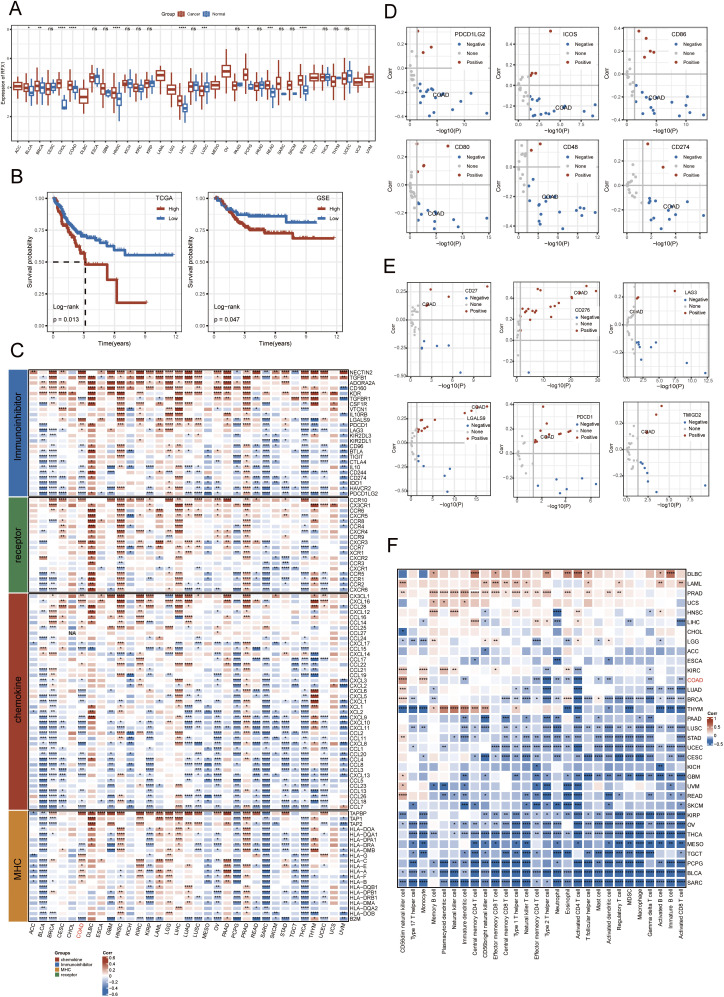
(**A**): RFX1 expression in pan-cancer, including associated normal paracancerous tissues. (**B**): The RFX1^high^ subgroup had the worst prognosis compared to the RFX1^low^ subgroup in both the training and validation sets. (**C**): Correlation of chemokines, immunosuppressive factors, MHC, and receptors with RFX1 in pan-cancer. (**D**): In colon cancer, PDCD1LG2, ICOS, CD86, CD80, CD48, and CD274 negatively correlated with RFX1 expression. (**E**): CD27, CD276, LAG3, LGALS9, PDCD1, and TMIGD2 were positively correlated with RFX1 expression in colon cancer. (**F**): Correlation of RFX1 with immune cell scores in pan-cancer. “*” indicates *p* < 0.05, “**” indicates *p* < 0.01, “***” indicates *p* < 0.001, “****” indicates *p* < 0.0001, and “ns” indicates no statistical significance (*p* ≥ 0.05)

**Figure A2:**
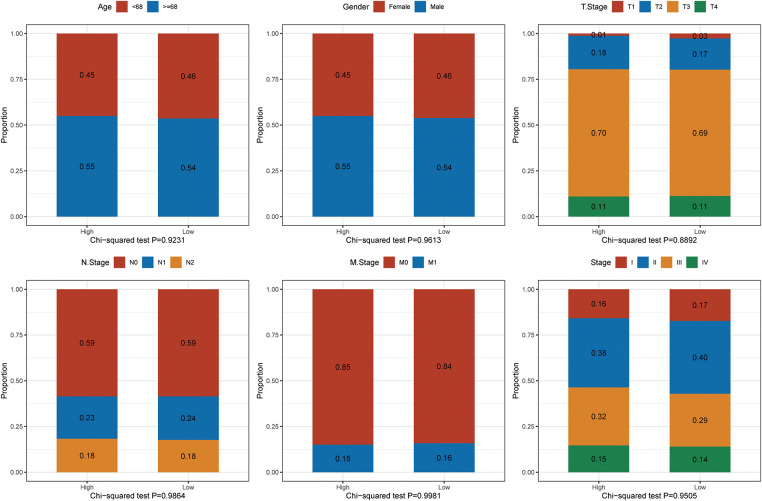
Comparison of key clinical variables—including age (median cutoff: 68 years), sex, stage, and others—between the high and low RFX1 expression groups

By comprehensively analysing the expression profile of RFX1 in pan-cancer, we found that RFX1 has higher expression in digestive tract tumors, including colon, cholangiocarcinoma and gastric cancer, compared to normal paraneoplastic tissues (Fig. 1A). In contrast, RFX1 expression was low in cancers such as cervical squamous cell carcinoma and lung adenocarcinoma (Fig. 1A). The differential pan-cancer expression of RFX1 prompted us to explore its prognostic value in digestive tract tumors, particularly colon cancer. In the TCGA dataset, based on the recurrence time and status, we took the best cutoff value (optimal cutoff value: 4.47854) for RFX1 expression values by R package “survminer” and divided the samples into two groups of high and low expression. Further, we plotted the KM curves for the high and low expression subgroups, with the high expression subgroup having a worse prognosis (Fig. 1B). To account for potential confounding factors in the survival analysis, we compared key clinical variables—including age (median cutoff: 68 years), sex, and tumor stage—between the high and low RFX1 expression groups using chi-square tests. The results showed no significant intergroup differences, indicating that these clinical features were evenly distributed (Fig. A2). Similarly, the RFX1 high expression subgroup has a worse prognosis in the GSE dataset. In short, RFX1 is higher in digestive tract cancers and linked to worse prognosis in colon cancer, highlighting its potential as a prognostic marker.

Characterising the immune landscape of RFX1 in pan-cancer facilitates the profiling of potential anti-RFX1 immunotherapies or the targeting of specific tumor markers. We obtained 122 marker genes for immunomodulators, including chemokines, immunosuppressive factors, MHC, receptors, and other characterisation dimensions, and explored their relevance to RFX1 in pan-cancer (Fig. 1C). The study suggests that in bladder, breast, pheochromocytoma, thyroid and endometrial cancers, RFX1 expression is negatively correlated with the four classes of immunomodulators mentioned above. In contrast, RFX1 was positively correlated with immunomodulators in hepatocellular carcinoma as well as prostate cancer. In colon cancer, RFX1 expression was mainly negatively correlated with chemokine-associated immunokines, such as CXCL10 and CXCL11. CXCL10 and CXCL11 are critical chemokines for cytotoxic T cell recruitment. Their suppression in RFX1-high tumors implies that RFX1 may help tumors evade immune surveillance by preventing effective T cell infiltration, consistent with an immunosuppressive phenotype. Further, we characterized the correlation between immune checkpoint-related genes and RFX1 in colon cancer. The results showed that PDCD1LG2, ICOS, CD86, CD80, CD48, and CD274 showed a significant exclusion situation from RFX1 expression in colon cancer (Fig. 1D). In contrast, CD27, CD276, LAG3, LGALS9, PDCD1, and TMIGD2 showed a highly enriched trend of expression with RFX1 in colon cancer (Fig. 1E). In addition, we assessed the degree of infiltration of each immune cell in the tumor immune microenvironment by ssGSEA based on the characteristic gene expression of the immune cells (Fig. 1F). The results showed that RFX1 showed a negative correlation with the infiltration of immune cells in most of the tumors, such as bladder cancer and sarcoma. However, in a few carcinomas, mainly represented by pancreatic cancer and acute myeloid leukaemia, RFX1 showed a highly positive correlation with the infiltration of each immune cell, with a tendency of significant enrichment. In colon cancer, type 2 T helper cells and activated CD4 T cells were highly negatively correlated with RFX1. While CD56 dim NK cells and monocytes were positively correlated with RFX1 expression. In summary, RFX1 is closely linked to immune modulation across cancers. In colon cancer, its high expression is associated with reduced T cell recruitment and altered immune checkpoint patterns, suggesting an immunosuppressive role in the tumor microenvironment.

### 3.3 Mutational Landscape of RFX1^***high***^ and RFX1^***low***^ Subgroups in Colon Cancer

**Figure 2:**
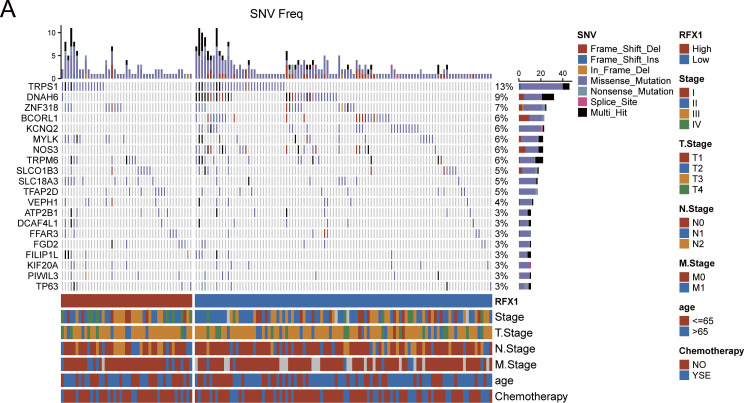

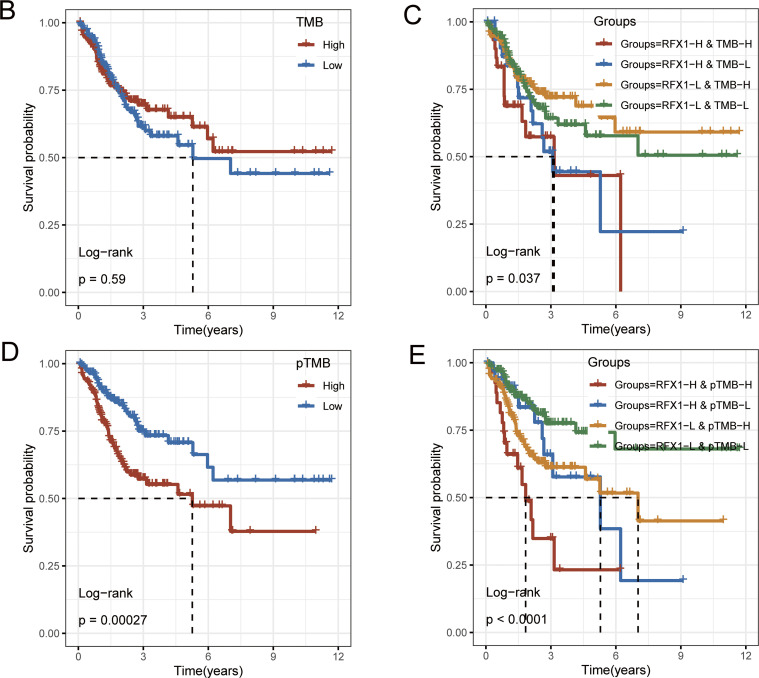
(**A**): Mutation landscape of the top 20 tumor driver genes with differential mutation frequencies in RFX1^high^ and RFX1^low^. (**B**): There was no significant difference in patient prognosis with TMB^high^ and TMB^low^ subgroups. (**C**): Combining RFX1 and TMB expression, the RFX1^low^/TMB^high^ subgroup suggests that patients have the best survival prognosis. (**D**): In the subgroups with pTMB^high^ and pTMB^low^, pTMB was a risk factor for poor patient prognosis. (**E**): Combining RFX1 and pTMB expression, the RFX1^high^/pTMB^high^ subgroup suggests that patients have the worst survival prognosis

**Figure A3:**
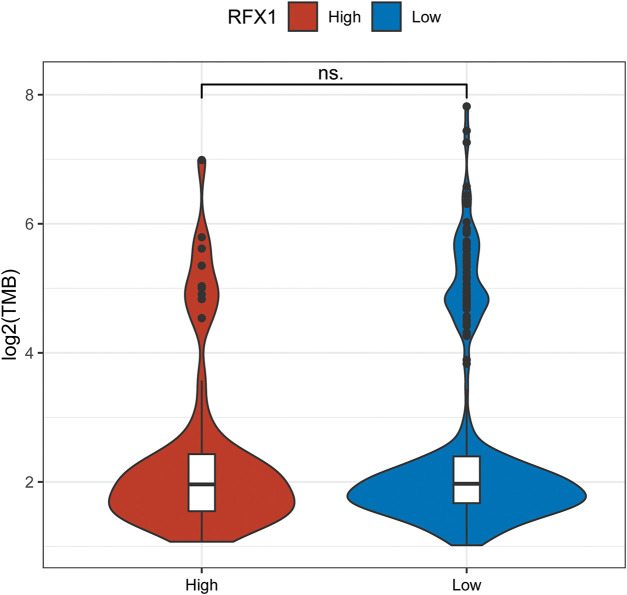
There was no significant difference in the expression of TMB in the RFX1^high^ and RFX1^low^ subgroups. “ns” indicates no statistical significance (*p* ≥ 0.05)

We identified 83 genes that differed significantly in RFX1^high^ and RFX1^low^ subgroups, and selected the differential genes with the highest mutation frequencies among them to plot a waterfall map (Fig. 2A). TRPS1 and DNAH6 had the highest mutation frequencies, 13% and 9%, respectively. And the top five genes including ZNF318, BCORL1 and KCNQ2 were mutated much more frequently in the RFX1^high^ subgroup than in RFX1^low^. Therefore, RFX1 may contribute to tumor progression by promoting the accumulation of specific somatic mutations and influencing genomic stability. Further, we compared the difference in the distribution of tumor mutant burden (TMB) in the two subgroups and found no significant difference in TMB (Fig. A3). In addition, we plotted KM curves after dividing into TMB^high^ and TMB^low^ based on the optimal cutoff value (optimal cutoff value: 2.894737), and again there was no significant difference (*p* = 0.59; Fig. 2B). Interestingly, when we combined the TMB subgroup with the RFX1 subgroup, the prognostic survival curves showed significant differences (*p* = 0.037; Fig. 2C). Among them, the RFX1^high^/TMB^high^ subgroup had the worst prognosis and the RFX1^low^/TMB^high^ subgroup obtained the best survival prognosis.

pTMB is a new metric characterizing the highly mutation-holding properties of tumor evolution and is not significantly associated with TMB [28]. Unlike traditional TMB, pTMB reflects the long-term retention of mutations that may be more relevant to tumor adaptability and therapy resistance. Our findings indicate that the pTMB^high^ subgroup possesses a greater capacity to delineate a notably dire prognosis in contrast to the TMB^high^ subgroup (*p* = 0.00027; Fig. 2D). Surprisingly, the RFX1^low^/pTMB^low^ subgroup and the RFX1^high^/pTMB^high^ subgroup had two distinct survival profiles, with the former having a better prognosis than the latter and being much more discriminatory than the predictive model combining RFX1 and TMB (*p* < 0.0001; Fig. 2E). The stronger association between pTMB^high^ and poor prognosis suggests that persistent genomic instability, rather than mutational quantity alone, drives worse clinical outcomes. These results further underscore the synergistic prognostic power of combining RFX1 expression with pTMB.

## 4 RFX1 Shapes Inflammatory TME and Participates in Crosstalk in Signalling Pathways

**Figure 3:**
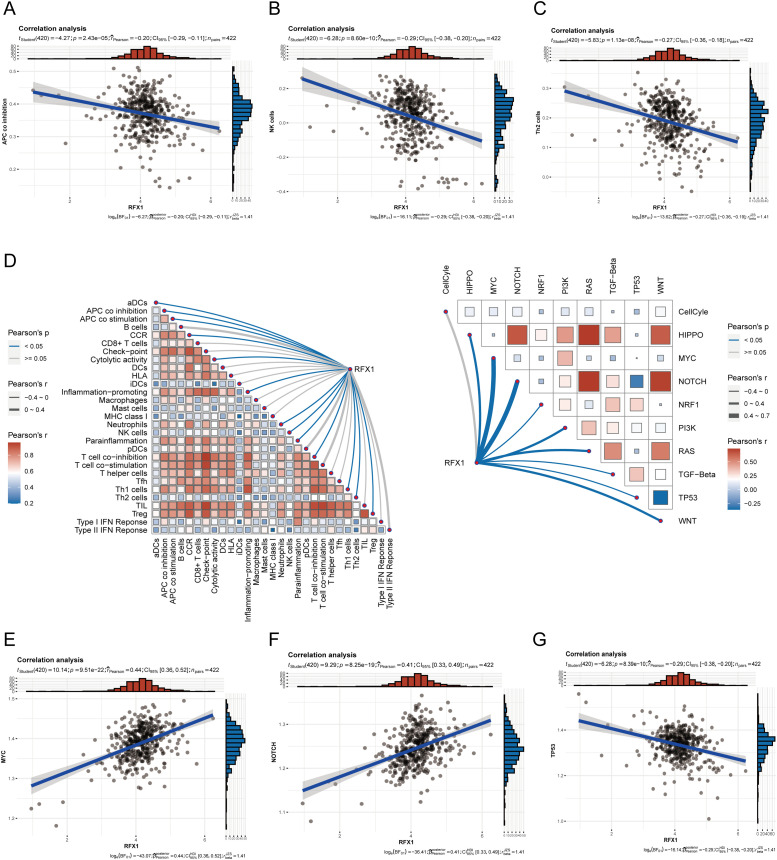
(**A–C**): In the tumor microenvironment, RFX1 expression was negatively correlated trend with the enrichment of APCs, NK cells, and Th2 cells. (**D**): Correlation of RFX1 with tumor immune microenvironment immune cell score and oncogenic pathway score is shown. (**E**–**G**): In the tumor microenvironment, RFX1 expression was positively correlated with the MYC and Notch pathways and a negative trend with the TP53 pathway

**Figure A4:**
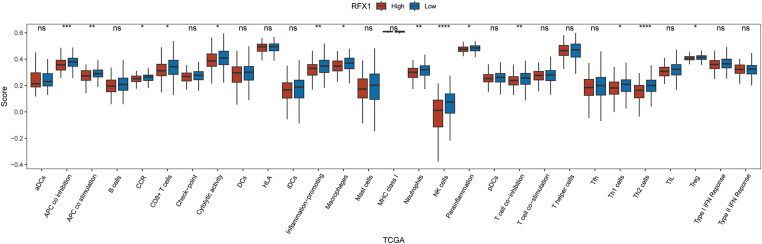
Differential expression of 29 immune cells in two subgroups, RFX1^high^ and RFX1^low^. “*” indicates *p* < 0.05, “**” indicates *p* < 0.01, “***” indicates *p* < 0.001, “****” indicates *p* < 0.0001, and “ns” indicates no statistical significance (*p* ≥ 0.05)

**Figure A5:**
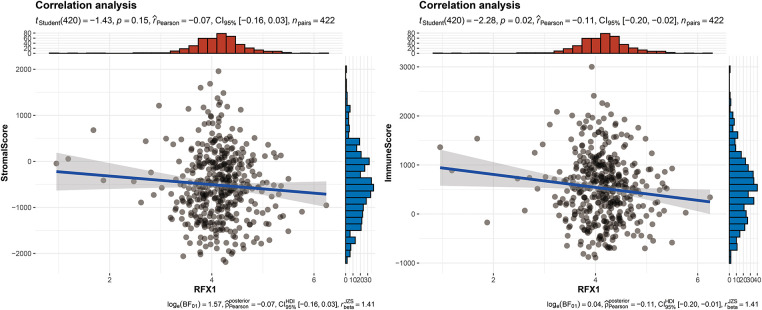
RFX1 expression was negatively correlated with both the stromal score (*p* < 0.01) and the immune score (*p* < 0.001)

**Figure A6:**
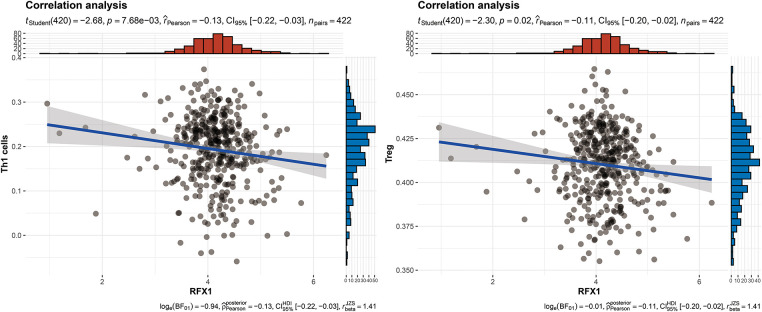
RFX1 expression showed a negative correlation trend with the enrichment of Th1 cells (*p* = 7.68 ∗ 10^−3^, R = −0.13) and Treg cells (*p* = 0.02, R = −0.11)

**Figure A7:**
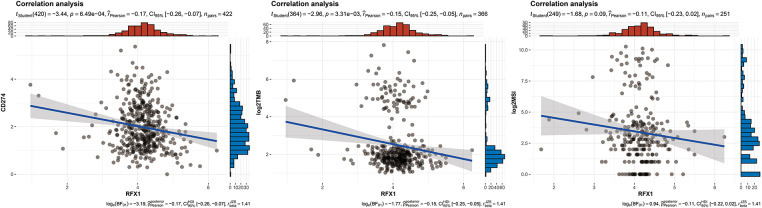
Correlation analysis between RFX1 expression and three well-established immune escape-related markers: PD-L1, MSI status, and TMB

**Figure A8:**
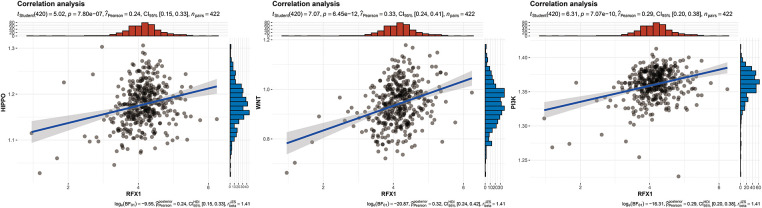
RFX1 expression showed a positive correlation trend with the enrichment of hippo pathway (*p* = 7.8 ∗ 10^−7^, R = 0.24), wnt pathway (*p* = 6.45 ∗ 10^−12^, R = 0.33), and PI3K pathway (*p* = 7.07 ∗ 10^−10^, R = 0.29)

Our study delved into the correlation between RFX1 and diverse immune cell populations within the intricate landscape of the immune microenvironment. Previously, we found that in colon cancer, RFX1 showed a negative trend in correlation with the expression of key chemokines (CXCL9 and CXCL10) that induce the recruitment of CD8^+^ T cells to the tumor microenvironment. Consistently, within the milieu of the immune microenvironment, the RFX1^low^ subgroup exhibited a notably higher infiltration of CD8^+^ T cells in comparison to the RFX1^high^ subgroup. It was found that the CXCR3-CXCL9/CXCL10 axis recruits CD8^+^ T cells and is dependent on CXCL9/CXCL10 as an “amplifier” of T cell recruitment to achieve T cell infiltration [37]. Interestingly, CXCR3 expression and recruitment were also significantly higher in the RFX1^low^ subgroup than in the RFX1^high^ subgroup. Thus, in the RFX1^high^ subgroup, a low degree of enrichment of the CXCR3-CXCL9/CXCL10 axis inhibited the infiltration of CD8^+^ T cells. In addition, Treg cells were also recruited by the CXCR3-CXCL9/CXCL10 axis, and the high expression of RFX1 significantly reduced Treg infiltration. These findings collectively suggest that RFX1 may act as a negative regulator of T cell trafficking into the tumor microenvironment by downregulating key chemokines and their receptors. The correlation analysis results revealed a negative association between RFX1 and the extent of APC (r = −0.20, *p* = 2.43 ∗ 10^−5^, n = 422), NK cell (r = −0.29, *p* = 8.6 ∗ 10^−10^, n = 422), Th1 (r = −0.13, *p* = 7.68 ∗ 10^−3^, n = 422), Th2 (r = −0.27, *p* = 1.13 ∗ 10^−8^, n = 422), and Treg cell (r = −0.11, *p* = 0.02, n = 422) enrichment (Figs. 3A–C and A4). These correlations indicate that RFX1 may broadly suppress both innate and adaptive immune cell infiltration. We next assessed the association between RFX1 expression and components of the tumor microenvironment, focusing specifically on stromal and immune infiltration. Correlation analysis showed that RFX1 expression was negatively associated with both stromal score (*p* < 0.01) and immune score (*p* < 0.001) (Fig. A5), suggesting that tumors with high RFX1 expression tend to have reduced stromal content and diminished immune cell infiltration. These findings reinforce the hypothesis that RFX1 may promote an immunosuppressive or “immune-cold” tumor microenvironment, potentially by interfering with immune recruitment or attenuating pro-inflammatory signaling. In addition, Th1 cells are involved in the anti-tumor immune response, whereas Th2 cells act as antagonists to Th1 cells. Surprisingly, RFX1 was negatively correlated with pro-inflammatory processes, CCR, cytolytic activity, parainflammation, and inhibited the progression of inflammation (Fig. A6). To further explore the relationship between RFX1 and immune escape, we analyzed its correlation with several established markers, including PD-L1 (CD274), MSI status, and TMB. RFX1 expression was significantly negatively correlated with PD-L1 (*p* < 0.001) and TMB (*p* < 0.01), suggesting that higher RFX1 levels may be associated with a reduced immune activation phenotype (Fig. A7). Although a similar negative trend was observed with MSI status, the correlation did not reach statistical significance (*p* > 0.05) (Fig. A7). These results indicate that RFX1 may contribute to the development of an immune-cold tumor microenvironment and play a role in immune evasion. Thus, in the complex tumor microenvironment, RFX1 appears to be a potential marker of tumor immune alterations, especially of the inflammatory microenvironment. However, we did not observe significant differences in RFX1 expression with DCs, Tfh, B cells, and IFN responses (Fig. 3D). At the level of signalling pathways, RFX1 expression was closely associated with Hippo, MYC (r = 0.44, *p* = 9.51 ∗ 10^−22^, n = 422), Notch (r = 0.41, *p* = 8.25 ∗ 10^−19^, n = 422), PI3K, TP53 (r = −0.29, *p* = 8.39 ∗ 10^−10^, n = 422) and WNT pathways (Figs. 3E–G and A8). Among them, the recruitment of MYC, Notch and WNT was significantly higher in the RFX1^high^ subgroup than in the RFX1^low^ subgroup, whereas TP53 was more significant in RFX1^low^ subgroup. This suggests that RFX1 may influence tumor biology not only through immune modulation but also via classical oncogenic signaling. The strong correlation with MYC and Notch signaling implies a role in promoting cell proliferation and stemness, whereas the inverse correlation with TP53 may indicate a suppression of tumor suppressor pathways.

## 5 Constructing Nomogram Models for Clinical Prediction

**Figure 4:**
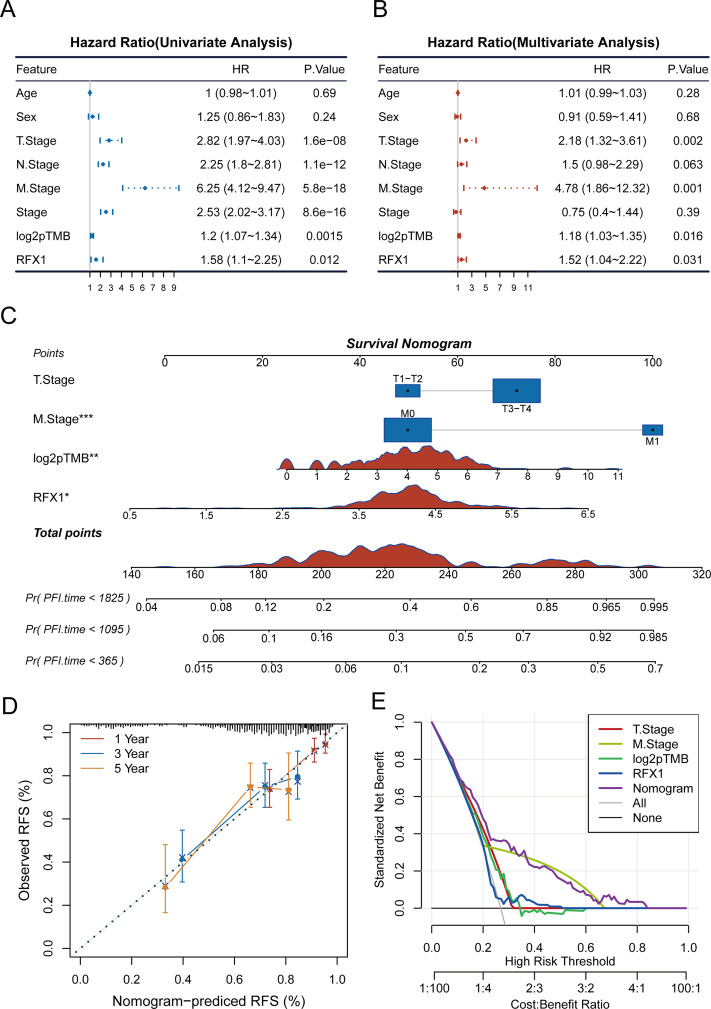
(**A**,**B**): Univariate and multivariate COX analyses of clinically relevant indicators, RFX1, and pTMB with tumor risk. (**C**): Construction of a nomogram model combining clinical indicators as well as RFX1 expression. (**D**,**E**): Nomogram model data correction and DCA analysis. “*” indicates *p* < 0.05, “**” indicates *p* < 0.01, “***” indicates *p* < 0.001

Previously, high RFX1 expression has been elucidated to be significantly associated with poorer patient prognosis, including cancer progression and immune microenvironment. To clarify whether RFX1 has clinical predictive efficacy, we performed univariate and multivariate COX analyses by combining age, sex, TNM stage, stage, log2pTMB, and other clinical characteristics (Fig. 4A,B). The results suggest that the gene RFX1 was significantly associated with prognosis in both univariate and multivariate analyses, while other characteristics such as T stage, M stage, and log2pTMB were equally significant. Therefore, combining the above-mentioned salient features associated with prognosis, we constructed a nomogram clinical prediction model to assess the overall survival of colon cancer patients (1, 3, and 5 years) (Fig. 4C). We used the R package “rms” (v6.7-1) as well as regplot (v1.2.2) to analyze and visualize the nomogram model, and performed a correlation analysis to verify its rigor and rationality (Fig. 4D). In addition, we performed a DCA analysis (Decision Curve Analysis) using the R package rmda (v1.6), but RFX1 did not perform as well as the other features (Fig. 4E).

## 6 RFX1 Predicts Immunotherapy Response and Drug Sensitivity

**Figure 5:**
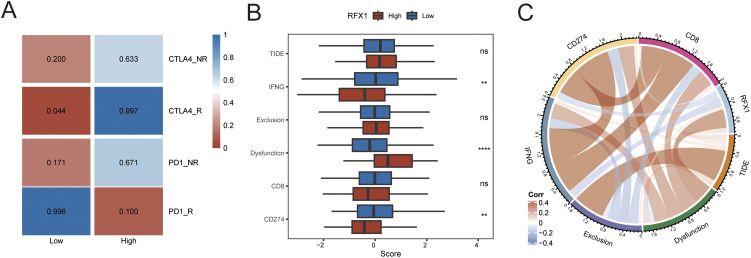

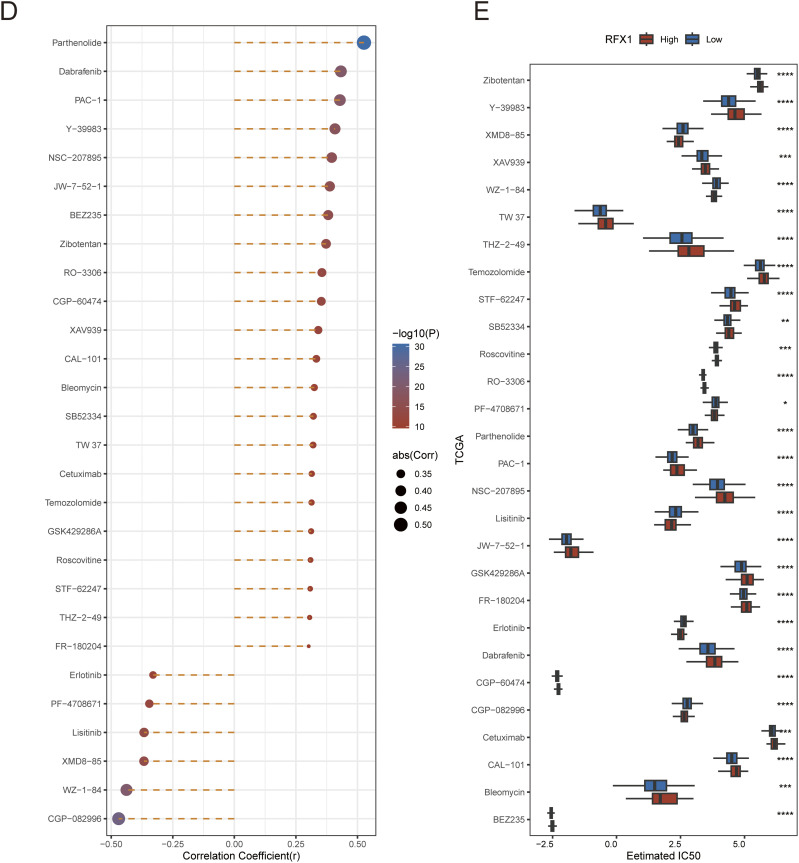
(**A**): Submap immunomapping of the TCGA dataset and GSE91061. (**B**): Analysis of differences in TIDE groupings for the TCGA dataset. (**C**): Correlation analysis of TIDE results for the TCGA dataset. **(D**): Correlation analysis of RFX1 with drug IC50. (**E**): Differential analysis of drug IC50 in RFX1^high^ and RFX1^low^. “*” indicates *p* < 0.05, “**” indicates *p* < 0.01, “***” indicates *p* < 0.001, “****” indicates *p* < 0.0001, and “ns” indicates no statistical significance (*p* ≥ 0.05)

**Figure A9:**
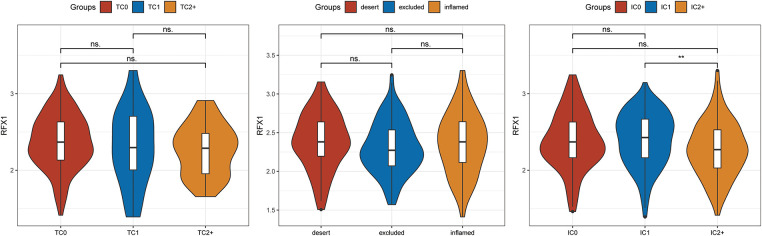
On the basis of immune status, tumors are classified as inflamed, with marked immune infiltration; excluded, with immune cells infiltrating into the interior of the tumor and enriched on the exterior of the tumor, and desert, with tumors almost devoid of immune infiltrating cells. TC: tumor cell positivity rate. TC = (number of tumor cells positive for PD-L1 membrane staining of any intensity/total tumor cells) × 100%. IC: tumor-associated immune cell positivity rate. IC = (number of tumor-associated immune cells positive for PD-L1 membrane staining of any intensity/total number of tumor-associated immune cells) × 100%. “**” indicates *p* < 0.01, and “ns” indicates no statistical significance (*p* ≥ 0.05)

To further elucidate the role of RFX1 in immunotherapy, we first evaluated the association between RFX1 and common immune checkpoint inhibitors in colon cancer. RFX1 was negatively correlated with BTLA expression, positively correlated with PD-1 and LAG3, and had no significant correlation with TIGHT. The GSE91061 dataset, derived from a phase II clinical trial (CA209-038), includes RNA-seq data from 109 tumor biopsies of 65 metastatic melanoma patients treated with anti-PD-1 therapy [38]. It comprises 51 baseline and 58 on-treatment samples collected approximately 12 weeks post-treatment. We mapped RFX1^high^ and RFX1^low^ subgroup expression profiles to another published dataset, GSE91061, which contains patients treated with CTLA4 as well as PD1, to predict RFX1 sensitivity to immunotherapy. In the TCGA dataset, there was a significant correlation between the expression profiles of the RFX1^low^ subgroup and the CTLA4_R-responsive group, predicting that patients in the RFX1^low^ subgroup are more sensitive to or more profitable in response to anti-CTLA4 immunotherapy (Fig. 5A). On the contrary, colon cancer patients with high RFX1 expression may not be suitable for implementing immune checkpoint inhibitor therapy. The reason for this is not only attributed to poorer sensitivity, but may also induce a higher incidence of progression. In addition, we did not find significant differences in RFX1 expression in the deserted phenotype, TC0 (tumor cells with the lowest PD-L1 values) and IC0 (immune cells with the lowest PD-L1 values) groups in the IMvigor210 cohort (Fig. A9). This may be attributed to the greater heterogeneity between tumor cells and the difficulty in mapping them to each other.

We applied the TIDE algorithm to predict response to immunotherapy in both RFX1^high^ and RFX1^low^ subgroups (Fig. 5B). It was found that TIDE did not differ in the high and low subgroups. Whereas IFNG and CD274 had higher scores in the RFX1^low^ subgroup, dysfunction had lower scores in the RFX1^low^ subgroup. In addition, despite the high correlation between IFNG and CD274, both showed a trend of negative correlation with RFX1, which is consistent with the previous results (Fig. 5C). In addition, for the therapeutic effect of individual drugs, we performed IC50 assessment using the R package pRRophetic and analyzed the correlation between drug IC50 and gene RFX1 expression. After thresholding |R| > 0.3 and *p* < 0.05, we obtained 28 drugs, of which 22 were significantly positively correlated and 6 were significantly negatively correlated (Fig. 5D,E). The IC50 of both CGP-082996 and WZ-1-84 was significantly negatively correlated with RFX1 expression, with lower sensitivity in the RFX1^high^ subgroup.

## 7 Expression and Clinical Significance of RFX1 in Colon Cancer

**Figure 6:**
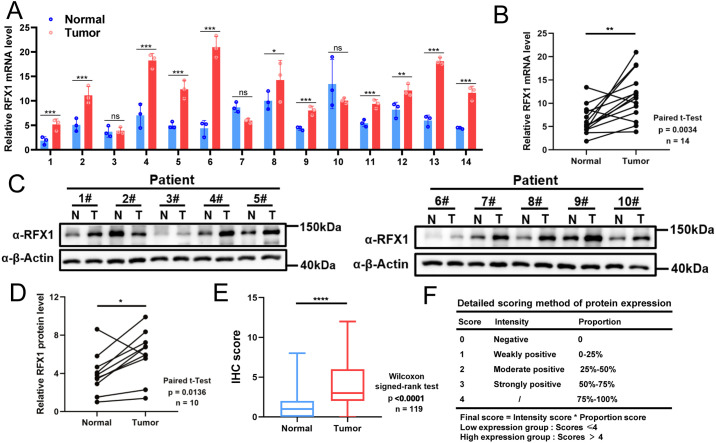

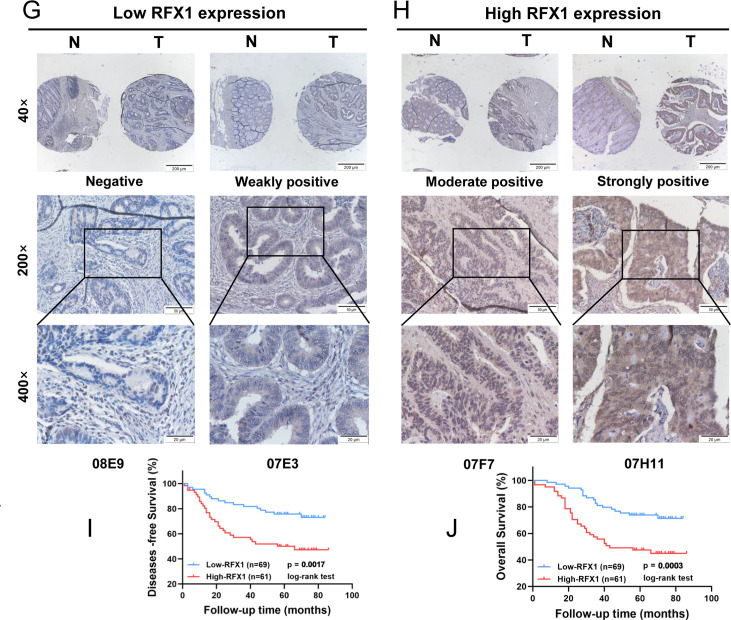
(**A**,**B**): mRNA analysis of 14 clinical tumor samples and normal tissue adjacent to the cancer. (**C**,**D**): Quantitative protein analysis of 10 clinical tumor samples and paraneoplastic normal tissue. (**E**): Differential score of RFX1 expression on immunohistochemical microarrays. (**F**): Immunohistochemical microarray scoring rules. (**G**,**H**): Immunohistochemical demonstration of RFX1 in RFX1^low^ and RFX1^high^ subgroups. (**I**,**J**): High RFX1 expression was significantly associated with lower DFS and OS. “*” indicates *p* < 0.05, “**” indicates *p* < 0.01, “***” indicates *p* < 0.001, “****” indicates *p* < 0.0001, and “ns” indicates no statistical significance (*p* ≥ 0.05)

Transitioning back to the clinical aspect of our study, we conducted an assessment of RFX1 expression in colorectal cancer and embarked on an exploration of its clinical relevance and significance. Through the application of qPCR to 14 pairs of colorectal cancer tissues and their respective normal tissue counterparts, we noted a substantial elevation in RFX1 mRNA expression levels within the colorectal cancer samples when juxtaposed with the normal tissues (Fig. 6A,B; *p* = 0.0034). Consistent findings were subsequently replicated through western blot analysis, where 10 pairs of colorectal cancer tissue specimens and their corresponding normal tissue counterparts demonstrated a parallel increase in RFX1 protein expression levels (Fig. 6C,D; *p* = 0.0136). In order to delve deeper into the clinical implications of RFX1 in the context of colorectal cancer, we conducted an immunohistochemical analysis using a colorectal cancer tissue microarray comprising 119 pairs of colorectal cancer and normal tissue samples. The results showed that the expression level of RFX1 was relatively low in normal intestinal epithelial cells, but significantly increased in tumor cells (Fig. 6E,F; *p* < 0.0001). We selected representative samples with high and low RFX1 expression from tissue microarrays for demonstration (Fig. 6G,H). In addition, we demonstrated the differential expression of RFX1 according to negative, low, moderate, and high trends at low and high magnification (Fig. 6G,H). Furthermore, Kaplan-Meier analyses unveiled that elevated levels of RFX1 expression served as an indicator of unfavorable prognosis in colorectal cancer. This association was linked to reduced overall survival (OS) (*p* = 0.0017) and disease-free survival (DFS) (*p* = 0.0003) among colorectal cancer patients (Fig. 6I,J).

## 8 RFX1 Promotes Invasive Metastasis of Colon Cancer *In Vitro*

**Figure 7:**
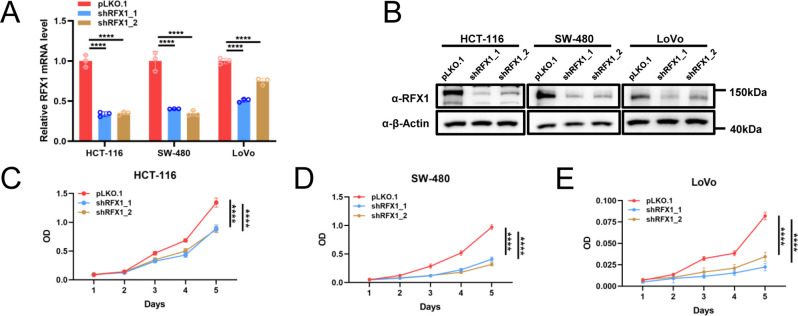

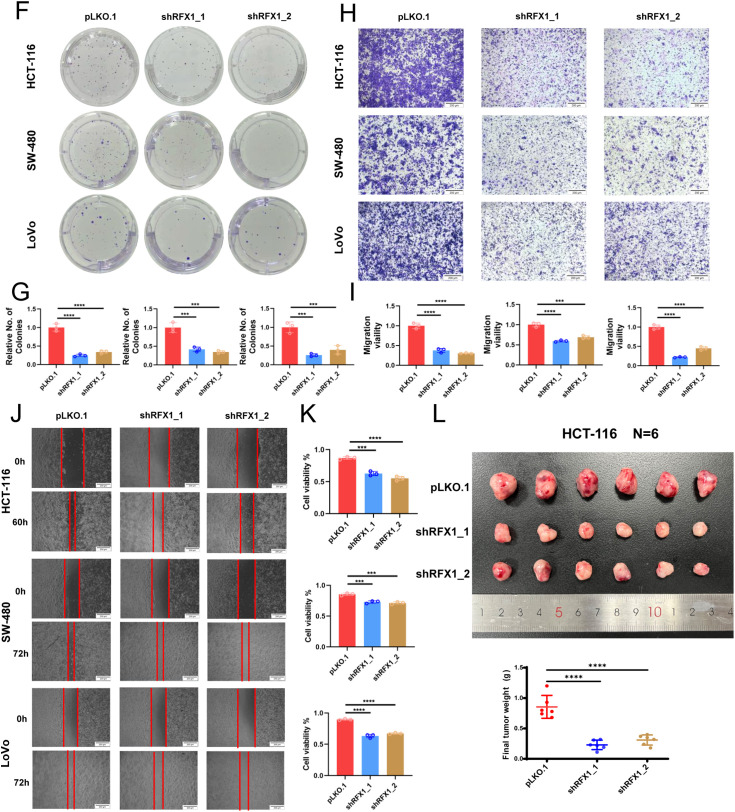
(**A**,**B**): Knockdown stable lines of RFX1 were constructed in HCT-116, SW-480 and LoVo and efficiency tests were completed. (**C**–**E**): CCK8 assay in HCT-116, SW-480 and LoVo. (**F**,**G**): Clone formation effects of RFX1 knockdown in HCT-116, SW-480 and LoVo. (**H**,**I**): The transwell effect of RFX1 knockdown stabilisation system in HCT-116, SW-480 and LoVo. (**J**,**K**): Scratch experiments on RFX1 knockdown stabilisation systems in HCT-116, SW-480 and LoVo. (**L**): Representative images of RFX1 knockdown HCT-116 xenograft tumours (n = 6). The weight of each xenograft was used for statistical analysis. “***” indicates *p* < 0.001, “****” indicates *p* < 0.0001

**Figure A10:**
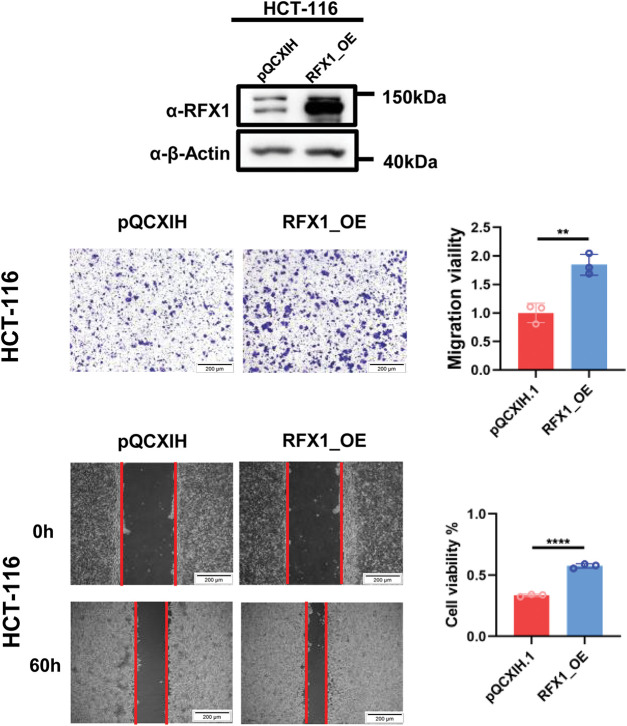
RFX1 overexpression stable lines were constructed at HCT-116 and transwell and scratch experiments were repeated. “**” indicates *p* < 0.01, “****” indicates *p* < 0.0001

In solid tumors such as cervical cancer and lung cancer, RFX1 can function as an oncogene through transcriptional regulation. Therefore, we constructed stable lines with knockdown of RFX1 in HCT-116, SW-480, and LoVo cell lines (Fig. 7A,B). The results of CCK-8 and clone formation assays showed that knockdown of RFX1 inhibited the proliferation ability (Fig. 7C–G). Meanwhile, the results of transwell and scratch assays in the three cell lines also supported that knockdown of RFX1 significantly inhibited the invasive and migratory ability of cells (Fig. 7H–K). To further validate the effect of RFX1 on colorectal cancer proliferation *in vitro*, we constructed another stable line overexpressing RFX1 in HCT-116 cell line. In contrast to the transwell and scratch results described above, overexpression of RFX1 in HCT-116 cells significantly promoted tumor invasion and metastasis (Fig. A10). Similar results were observed in *in vivo* xenograft trials (Fig. 7L).

## 9 Discussion

The RFX protein family encompasses a group of evolutionarily conserved DNA-binding proteins with integral roles in a myriad of critical cellular growth and developmental processes. Within this family, RFX1 stands out as a ubiquitously expressed transcription factor, exhibiting dual functionality that encompasses both the activation and repression of target genes [39]. In particular, the exploration of its transcriptional regulatory mechanisms in a variety of tumor cells is still at a preliminary stage. A cohort study including 124 patients with large HCC and 71 patients with small hepatocellular carcinomas found that reduced RFX1 in hepatocellular carcinoma tissues predicted a poor prognosis, as well as a high risk of recurrence [40]. And it was verified by *in vitro* experiments that knockdown of RFX1 facilitated the decreased expression of E-calmodulin as well as the increased expression of waveform proteins, which promoted the EMT and invasion of hepatocellular carcinoma cells [40]. RFX1 forms DNA-binding structural domains and functionally independent dimerization structural domains through affinity for HLA class II X-box promoter elements. This structure provides evidence for the involvement of RFX1 in enhancer I function in HepG2 hepatoma cells [41]. In its role as a transcriptional repressor, RFX1’s transcriptional activity in undifferentiated HL60 cells hinges on the presence of Myc intron-binding protein 1 [42]. Type I collagen is composed of three polypeptide chains co-transcribed by COL1A1 and COL1A2, each dependent on a coordinately regulated promoter. In cancer cells, methylation of the first exon of COL1A2, which is located at the regulator of the X-box (RFX) locus (at −1 to +20), is associated with increased RFX1 binding and decreased collagen transcription [43]. RFX1/HDAC1 inhibition of the type I collagen α2 chain COL1A2 promoter was enhanced when the CpG site within the RFX common motif was methylated [44]. Similarly, COL1A2 promoter activation was repressed by RFX1-recruited mSin3A [44]. In glioblastoma, RFX1 was shown to negatively regulate the self-renewal capacity of glioma stem cells through direct FGF1 suppression, and to inhibit tumor invasiveness via downregulation of CD44 [45]. Additionally, in breast cancer cells, RFX1 participates in the activation of the SHP1 promoter in response to serum stimulation, thereby attenuating cell proliferation through ERK pathway suppression [8]. These findings suggest that in certain tumor contexts, RFX1 acts as a transcriptional brake that limits oncogenic signaling and tumor progression.

Recently, Issac et al. reported for the first time that after exposure to bexarotene, binding of the RFX1 promoter to RXRα inhibited multidrug resistance in NT2 cells and induced cell sensitivity to cisplatin [16]. Within the context of glioblastoma, RFX1 appears to exert a potential negative regulatory influence on the self-renewal capacity of glioblastoma stem cells. This regulation is achieved through its binding to the cis-elements of the F1B promoter, consequently modulating the expression levels of FGF-1B and FGF1 [46]. Recent studies have confirmed that RFX1 directly reduces CD44 expression and inhibits glioblastoma invasion, suggesting the importance of RFX1 in tumor physiology [47]. It has been hypothesized that the RFX1 binding site is also present in the promoter of the human kinesin family member 3A gene, and that inhibition of this transcriptional process could be beneficial in suppressing invasive breast cancer [48]. Recent genomic analyses have identified conserved RFX1-binding motifs within the promoter region of Vangl2, implicating RFX1 as a potential transcriptional regulator of this gene [49]. Vangl2 is a core component of the non-canonical Wnt/planar cell polarity (PCP) signaling pathway, which plays a critical role in controlling cellular polarity and motility. Dysregulation of this pathway has been closely associated with tumor progression and metastasis. In particular, Vangl2 has been shown to promote the migration and invasion of colorectal cancer cells by activating the fibronectin/integrin β1/FAK signaling cascade through DVL2. These findings suggest that RFX1 may contribute to cancer progression and immune remodeling by upregulating Vangl2 expression, thereby enhancing non-canonical Wnt/PCP signaling and promoting a pro-metastatic tumor microenvironment. It is worth noting that the mechanism of self-inhibition of RFX1 may depend on the negative regulation of self-inhibition by recruiting the serine/threonine protein phosphatase catalytic subunit (PP1c) to bind to its own promoter [50].

In addition to regulating cellular events at the transcriptional level leading to progression of the tumor state, epigenetic modifications of RFX1 underlie the maintenance of tumor stem cell inheritance. Different subpopulations of DNA methylation patterns in adult gliomas revealed that RFX1 in low-grade astrocytes, oligodendrocytes, and early-onset glioblastoid tumors showed a highly methylated pattern that promoted the expression of the methyltransferase enhancer of zeste human homologue 2 and was associated with improved survival [51]. Ritchie et al. found that the 3’UTR of RFX1 is a target of miR-92 and that downregulation of RFX1 on this basis may induce upregulation of PCNA [52]. SNHG17 has been shown to be an oncogene in HCC and is involved in cell proliferation and invasion. RFX1 is regulated by miR-3810-3p, whose upstream SNHG17 contributes to HCC progression through the miR-3180-3p/RFX1 axis [53].

Besides in tumors, RFX1 is involved in its transcriptional mechanism in coronary atherosclerosis and systemic lupus erythematosus. In patients with coronary atherosclerosis, deletion of RFX1 activates TLR4 and is involved in disease progression through histone modifications that further activate CD14^+^ monocytes [54]. Similar to coronary atherosclerosis, decreased RFX1 expression in CD4^+^ T cells from patients with systemic lupus erythematosus induces histone H3 acetylation and reduces DNA methylation and H3K9 trimethylation, leading to IL-17A overexpression [55]. Finally, RFX1 protein expression is degraded in the proteasome pathway mediated by E3 ligase, STIP1 homology, and U-box-containing protein 1 (STUB1) polyubiquitination [56]. STUB1 overexpression in patients activates CD70 and CD11a in T cells compared to healthy subjects, and its DNA demethylation and histone hyperacetylation contributes to the development of autoreactivity and autoantibody hyperstimulation in CD4^+^ T cells in patients with erythematous lupus. In contrast, RFX1 enhances DNA methylation and histone acetylation in CD4^+^ T cells by recruiting the co-repressors DNMT1 and HDAC1 to the CD11a and CD70 promoters and inhibits their expression. Therefore, increased RFX1 expression is favorable for reducing the progression of lupus erythematosus by inhibiting T cell reactivity [57].

Cancer immunotherapy has become a landmark breakthrough that has transformed the quality of survival for cancer patients, but a large proportion of bowel cancer patients still struggle to respond effectively to immune checkpoint inhibitors. Currently, clinical biomarkers including PD-L1 and microsatellite instability are limited by the difficulty of balancing sensitivity and specificity. Remarkably, although SubMap analysis suggested that patients in the RFX1-low subgroup may be more sensitive to anti-CTLA-4 therapy, TIDE analysis did not reveal a significant difference in immunotherapy response between the RFX1 subgroups. This discrepancy may be attributed to the distinct analytical frameworks of the two tools: SubMap evaluates transcriptomic similarity to known responders, capturing pathway-level activation, while TIDE focuses on modeling immune dysfunction and exclusion mechanisms. These tools provide complementary perspectives, and further validation in immunotherapy-treated colon cancer cohorts is needed to confirm the predictive relevance of RFX1. In this context, tumor mutation burden is gradually coming to the attention of clinical scientists. For example, in patients with non-small cell lung cancer treated with nivolumab and ipilimumab, high TMB was strongly associated with progression-free survival independent of PD-L1. In addition, TMB has shown excellent predictive results in both bladder cancer and head and neck tumors. However, it is prudent to note that there is still a lack of uniform standardised evaluation and consensus on TMB in pan-cancer, especially the judgment of the critical value of high and low TMB in different tumors. The pTMB describes the sum of mutations in single-copy regions and mutations for which multiple copies are present in each cell [28]. In the microenvironment of cancer immunotherapy, where tumor persistent mutations are retained as the tumor progresses, high levels of pTMB also predicted progression of the microenvironment towards an inflammatory environment. We did not observe a difference in TMB between the RFX1^high^ and RFX1^low^ subgroups. Interestingly, TMB^high^ and TMB^low^ showed better predictive power when combined with RFX1 instead. High RFX1 expression continued to represent a worse prognosis, while it seemed to be independent of TMB expression. Compared to the ambiguity of TMB, pTMB showed a more robust and sensitive prognostic efficacy, and pTMB^high^ predicted a poorer prognosis. Surprisingly, when pTMB and RFX1 were combined, the pTMB^high^/RFX1^high^ subgroup had the worst prognosis, whereas the pTMB^low^/RFX1^low^ subgroup had the best prognosis. Moreover, the RFX1 high-expression subgroup not only shaped the inflammatory tumor microenvironment, but also correlated significantly with the IC50 of most drugs. Sorafenib-added RFX1 inducer SC-2001 was reported to be effective in ameliorating chemoresistance in a sorafenib-resistant HCC xenograft mouse model [58]. Further, by combining the expression of RFX1, pTMB and some clinical indicators, we developed a nomogram clinical prediction model to evaluate its effectiveness in actual clinical prediction. We found that M stage, log2pTMB and RFX1 were all statistically significant in this model and showed good predictive efficacy.

While our study provides substantial insights into the immunogenomic and prognostic role of RFX1 in colorectal cancer, several limitations remain. First, although we have performed both *in vitro* and *in vivo* functional assays confirming the oncogenic role of RFX1 in tumor progression, the specific immunomodulatory mechanisms–such as its regulation of immune cell recruitment or immune evasion pathways–were not directly addressed and require further mechanistic studies. Second, as the majority of our data were derived from retrospective public datasets, prospective validation in larger and independent clinical cohorts is still necessary. Finally, although we observed significant correlations between RFX1 expression and immunotherapy response indicators, its predictive utility needs to be confirmed in actual patient cohorts receiving immune checkpoint blockade. These aspects will be the focus of our future work.

To further validate the role of RFX1 in colorectal cancer, particularly in immune modulation and response to immunotherapy, future studies should focus on more in-depth mechanistic investigations. Specifically, establishing RFX1 knockdown or overexpression models in colorectal cancer cell lines, followed by co-culture with immune cells such as CD8^+^ T cells or macrophages and performing immune phenotyping, could help clarify its regulatory effects on immune cell recruitment and activation. For *in vivo* validation, immune-competent mouse models, such as syngeneic tumor models or humanized mice, treated with immune checkpoint inhibitors (e.g., anti-PD-1/PD-L1), will be essential to assess whether RFX1 expression influences therapeutic efficacy. Additionally, integrating ChIP-seq and RNA-seq analyses to systematically identify downstream targets and signaling pathways regulated by RFX1 will provide further insights into its mechanistic role in tumor-immune interactions and immune evasion. These studies will be critical in defining the functional significance of RFX1 and supporting its potential as a clinically actionable immunotherapeutic target in colorectal cancer.

## 10 Conclusion

Undeniably, as an oncogene, RFX1 is highly expressed in colon cancer and has significant prognostic value. We characterized the immune landscape, mutational features, shaping inflammatory features, immunotherapy and drug sensitivity of RFX1 in colon cancer. On this basis, we constructed a nomogram clinical prediction model in conjunction with pTMB and verified its clinical value after *in vitro* experiments to reveal its significance for survival within 5 years. The study of RFX1 in colon cancer is still in the preliminary stage, especially transcriptional regulation and epigenetic modifications, which may determine its pleiotropic characteristics. And the pleiotropic nature of RFX1 may shape its multifaceted roles in several key tumor signalling pathways. In the future, the reprogramming effect of RFX1 may facilitate the transformation of malignant stem cell populations into differentiated cells, reversing their responsiveness to classical drugs.

## Data Availability

The datasets used during the current study are available from the corresponding authors on reasonable request.

## Abbreviations

AD: Activation domain
DBD: DNA-binding domain
ICIs: Immune checkpoint inhibitors
pTMB: Persistent tumor mutational burden
RFX1: Regulatory factor X1
TMB: Tumor mutation burden
TCGA: The cancer genome atlas
